# Correlation between the Pharyngeal Airway Space and Head Posture after Surgery for Mandibular Prognathism

**DOI:** 10.1155/2015/251021

**Published:** 2015-04-22

**Authors:** Chun-Ming Chen, Steven Lai, Ker-Kong Chen, Huey-Er Lee

**Affiliations:** ^1^Department of Oral and Maxillofacial Surgery, Kaohsiung Medical University Hospital, Kaohsiung Medical University, No. 100, Shih-Chuan 1st Road, Kaohsiung 807, Taiwan; ^2^Department of Conservative Dentistry, Kaohsiung Medical University Hospital, Kaohsiung Medical University, No. 100, Shih-Chuan 1st Road, Kaohsiung 807, Taiwan; ^3^Graduate Institute of Dental Sciences, College of Dental Medicine, Kaohsiung Medical University, No. 100, Shih-Chuan 1st Road, Kaohsiung 807, Taiwan

## Abstract

*Purpose*. The aim of this study was to determine the correlation between the pharyngeal airway space and head posture after mandibular setback surgery for mandibular prognathism. *Materials and Methods*. Serial lateral cephalograms of 37 patients with mandibular prognathism who underwent intraoral vertical ramus osteotomy (IVRO) were evaluated before (T1) and immediately (T2), between 6 weeks and 3 months (T3), and more than 1 year (T4) after surgery. Paired *t*-tests and Pearson's correlation analysis were used to evaluate the postoperative changes in all cephalometric parameters, including the mandible, hyoid, head posture (craniocervical angle), and pharyngeal airway space. *Results*. The mandible and hyoid were set back by 12.8 mm and 4.9 mm, respectively, at T2. Furthermore, the hyoid showed significant inferior movement of 10.7 mm, with an 8 mm increase in the tongue depth. The upper oropharyngeal airway (UOP) shortened by 4.1 mm, the lower oropharyngeal airway (LOP) by 1.7 mm, and the laryngopharyngeal airway by 2 mm. The craniocervical angle showed a significant increase of 2.8°. UOP and LOP showed a significant correlation with the craniocervical angle at T2 and T4. *Conclusions*. Our findings conclude that the oropharyngeal airway space is significantly decreased and correlated with a change in the head posture after mandibular setback surgery.

## 1. Introduction

Görgülü et al. [1] found that the root of the tongue was positioned more inferiorly and anteriorly in patients with Class III malocclusion than in those with Class I malocclusion. Abu Allhaija and Al-Khateeb [2] investigated the uvuloglossopharyngeal dimensions in subjects with different anteroposterior jaw relationships and found that the hyoid bone was more anteriorly positioned in Class III subjects than in Class I and Class II subjects. These findings suggest that the hyoid bone is located posteriorly and superiorly in Class II patients and anteriorly and inferiorly in Class III patients. Battagel et al. [3] studied patients with obstructive sleep apnea and Class II malocclusion and found that the hyoid bone position was more backward, which comparatively narrowed the upper airway.

El and Palomo [4] reported that the mandibular position with respect to the cranial base affected the oropharyngeal airway volume, which was smaller in Class II patients than in Class I and Class III patients. After orthognathic surgery, the mandibular bone, tongue, hyoid bone, and epiglottis move backward, resulting in narrowing of the pharyngeal airway space [5]. Therefore, patients require postural adaptations to get accustomed to a narrow airway. The aim of the present study was to investigate the hypothesis that there is no significant correlation between changes in the head posture and a decrease in the pharyngeal airway space after mandibular setback surgery.

## 2. Materials and Methods

A total of 37 patients (26 women and 11 men; mean age, 20.8 years) with Angle Class III malocclusion accompanied by mandibular prognathism who underwent modified bilateral intraoral vertical ramus osteotomy (IVRO) without any other surgery were recruited from the Oral and Maxillofacial Surgery Department of Kaohsiung Medical University Chung-Ho Memorial Hospital. A single surgeon had operated on all patients. Other inclusion criteria were as follows: availability of follow-up radiographs for at least 1 year, absence of facial asymmetry, absence of trauma or other congenital craniofacial abnormalities, and cessation of mandibular growth and development. All patients consulted both an orthodontist and an oral and maxillofacial surgeon, and preoperative orthodontic treatment was initiated after careful analysis and evaluation of all data and the preparation of a detailed and step-by-step treatment plan.

Following surgery, an occlusal wafer splint was used to fix the mandibular bone with additional maxillomandibular fixation, although skeletal and bone segment fixation was not performed. Generally, intermaxillary fixation was removed 6 weeks after surgery. Lateral cephalograms to examine the pharyngeal airway depth and positional changes in the mandibular bone, head, tongue, and hyoid bone were obtained before (T1) and immediately (T2), between 6 weeks and 3 months (T3), and 1 year ( T4) after surgery. The head posture of each patient was controlled by the laser light of the panorex machine. For midsagittal and horizontal planes, the patient's head was kept straight, with the Frankfort plane parallel to the laser. The panorex machine was operated by a single and experienced radiologist at all time points.

**Figure 1:**
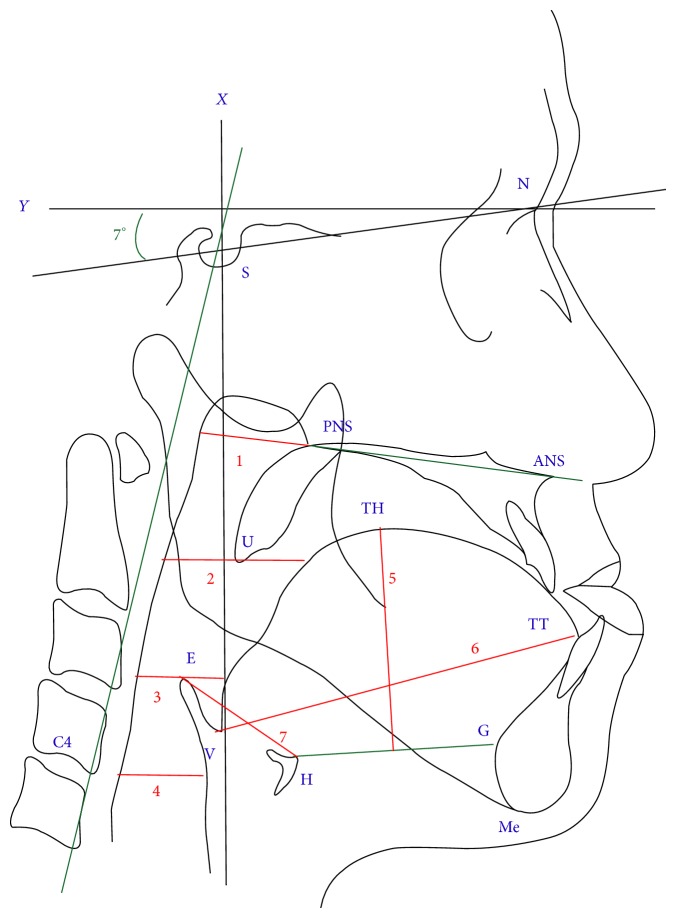
S: sella; N: nasion; Me: menton; ANS: anterior nasal spine; PNS: posterior nasal spine; G: most prominent point on the posterior border of the mandibular symphysis; UT: tip of uvula; C4: inferoanterior point on the fourth cervical vertebra; TT: tip of the tongue; E: most superior point on the epiglottis; V: vallecula epiglottica; H: most superior and anterior point on the hyoid bone; and TH: highest point on the tongue (greatest vertical distance to the H-G line). Green lines: C4S line, ANS-PNS line, and H-G line. Red lines: 1: NSP: nasopharyngeal airway, 2: UOP: upper oropharyngeal airway, 3: LOP: lower oropharyngeal airway, 4: LGP: laryngopharyngeal airway, 5: tongue depth, 6: tongue length, and 7: H-E length.

The reference points and definitions used in this study were as follows: S: sella; N: nasion; Me: menton: most inferior point on the mandibular symphysis; ANS: anterior nasal spine; PNS: posterior nasal spine; G: most prominent point on the posterior border of the mandibular symphysis; UT: tip of uvula; C4: inferoanterior point on the fourth cervical vertebra; TT: tip of the tongue; E: most superior point on the epiglottis; V: vallecula epiglottica; H: most superior and anterior point on the hyoid bone; and TH: highest point on the tongue (greatest vertical distance to the H-G line). The following reference lines were used (Figure 1): *X*-axis, constructed by drawing a line through the nasion, 7° above the SN line; *Y*-axis, constructed by drawing a line through S, perpendicular to the *X*-axis; and C4S line. The measurement parameters included the following: NSP: nasopharyngeal airway (ANS-PNS plane intersecting the pharyngeal wall); UOP: upper oropharyngeal airway (distance between the horizontal plane through the UT, intersecting the posterior border of the tongue, and posterior pharyngeal wall); LOP: lower oropharyngeal airway (distance between the horizontal plane through E, intersecting the posterior border of the tongue, and posterior pharyngeal wall); LGP: laryngopharyngeal airway (horizontal plane through C4, intersecting the pharyngeal wall); H-E: distance between H and E; tongue length (distance between V and TT); tongue depth (greatest vertical distance through TH to the H-G line); and craniocervical angle (angle between the C4S line and SN line). All serial cephalograms were evaluated twice by author (Chun-Ming Chen), and if the difference between the two values for any point or angle exceeded 0.5 mm or 1°, respectively, the point or angle was registered a third time. The third registration was compared with the others, and the mean of the two closest values was selected for analysis.

Changes in the abovementioned measurements at every time point were quantified and statistically analyzed using paired *t*-tests, which included mean values, standard deviations, and 95% confidence intervals. Pearson's correlation coefficient was used to analyze significant differences in the pharyngeal airway depth and position of the mandibular bone, head, tongue, and hyoid bone. The hypothesis is that there is no significant correlation between changes in the head posture and a decrease in the pharyngeal airway space after mandibular setback surgery.

## 3. Results

Compared with the positions at T1, significant changes were observed at T2 (Table 1). Me, TH, H, and C4 moved backward by 12.8, 7.1, 4.9, and 5.6 mm, respectively; TH moved upward by 2.7 mm; and H moved downward by 10.7 mm. UOP shortened by 4.1 mm, LOP by 1.7 mm, and LGP by 2 mm. The tongue depth increased by 8 mm, and the craniocervical angle (C4-SN) increased by 2.8° (Table 2). All parameters showed significant differences between T1 and T2.

Compared with the positions at T2, significant changes were observed at T3 (Table 1). Me moved backward by 3.3 mm, TH moved forward by 6 mm, and H moved upward by 8.8 mm. NSP shortened by 1.6 mm, LOP shortened by 1.7 mm, LGP increased by 2 mm, and HE decreased by 3.1 mm. The tongue depth and length decreased by 4.6 and 3.2 mm, respectively (Table 2). All parameters showed significant differences between T2 and T3. Compared with these changes at T3, further significant changes were observed at T4; Me moved forward by 3.1 mm and TH upward by 2.6 mm, and HE increased by 1.2 mm.

The following significant changes were observed at T4 relative to the positions at T1 (Table 1): Me moved backward by 12.9 mm, TH moved backward by 2.7 mm and upward by 4.6 mm, and H moved backward by 4.8 mm. UOP shortened by 4.7 mm, LOP decreased by 2.4 mm, and the tongue depth and length increased and decreased by 5 and 2.5 mm, respectively (Table 2). With regard to postoperative stability, the following changes were observed at T4 relative to those at T2 (Table 1). Me moved backward by 0.1 mm and upward by 1.9 mm, with no significant difference, while TH moved forward by 4.4 mm and H moved upward by 9 mm, showing significant change. NSP increased by 1.2 mm, HE decreased by 1.9 mm, and the tongue depth and length decreased by 3 and 3.2 mm, respectively (Table 2); all these parameters showed a significant difference.

Using the preoperative (T1) lengths of the pharyngeal airway spaces (NSP, UOP, LOP, and LGP) as baseline, Pearson's analysis was used to study the correlation between the immediate postoperative changes and the lengths of the pharyngeal airway spaces (Table 3). The length of NSP was significantly correlated with changes in TH (*r* = −0.328, *p* < 0.05), while the length of UOP was significantly correlated with horizontal movement of H (*r* = 0.420, *p* < 0.01) and C4 (*r* = −0.577, *p* < 0.01), changes in the C4-SN angle (*r* = 0.647, *p* < 0.01), and the length of LOP (*r* = 0.610, *p* < 0.01) and LGP (*r* = 0.360, *p* < 0.05). The length of LOP was significantly correlated with horizontal movement of C4 (*r* = 0.476, *p* < 0.01), changes in the craniocervical angle (*r* = 0.504, *p* < 0.01), and the lengths of UOP and LGP (*r* = 0.445, *p* < 0.01). The length of LGP was also significantly correlated with the lengths of UOP and LOP.

Using the lengths of the pharyngeal airway spaces (NSP, UOP, LOP, and LGP) as baseline, Pearson's analysis was used to study the correlation (Table 4). The length of NSP was significantly correlated with that of UOP (*r* = 0.332, *p* < 0.05). The length of UOP was significantly correlated with horizontal movement of H (*r* = −0.392, *p* < 0.05) and C4 (*r* = −0.492, *p* < 0.01), changes in the craniocervical angle (*r* = 0.368, *p* < 0.05), the lengths of NSP, LOP (*r* = 0.484, *p* < 0.01), and LGP (*r* = 0.405, *p* < 0.05), and changes in the tongue length (*r* = 0.503, *p* < 0.01). The length of LOP was significantly correlated with horizontal movement of C4 (*r* = −0.378, *p* < 0.05) and vertical movement of H (*r* = −0.346, *p* < 0.05), changes in the craniocervical angle (*r* = 0.407, *p* < 0.05), and the length of UOP. The length of LGP was significantly correlated with horizontal movement of Me (*r* = 0.487, *p* < 0.01), changes in the craniocervical angle (*r* = 0.347, *p* < 0.05), and the length of UOP and tongue depth (*r* = −0.345, *p* < 0.05). The decrease of oropharyngeal airway space is significantly correlated with postoperative change in the head posture. Therefore, the hypothesis of present study was rejected.

## 4. Discussion

The pharynx comprises three parts, which separate by soft palate and upper top of epiglottis cartilage, from the upper to lower end: nasopharynx, oropharynx, and laryngopharynx. The tongue is located on inner side of the mandible. The root of tongue connects the hyoid bone, soft palate, and pharynx by the hyoglossal and genioglossal muscles, glossopalatine arch, and superior pharyngeal constrictor muscle, respectively [6, 7]. The study of anatomical structures has revealed that structural changes in the airway can occur between the soft palate and epiglottis after mandibular setback surgery. The primarily affected structures include the soft palate, posterior edge of the tongue, epiglottis, and cervical vertebra. The strength of the present study was that changes in these structures were evaluated to determine the actual postoperative airway conditions. However, the lack of use of computed tomography to present 3D volumetric data remains a limitation.

Immediately after mandibular setback surgery (T2), the hyoid bone accounted for approximately 38% of the mandible setback, while TH accounted for 55%. However, in the vertical direction, the hyoid bone accounted for approximately 7 times of the mandible downward movement and TH point for 1.7 times upward. Therefore, alternative positions of the hyoid bone and tongue could counteract the oppression of mandibular setback. Furthermore, the fourth vertebra showed significant backward movement, accounting for approximately 44% of the mandibular setback. These changes were possibly related to postoperative airway narrowing and natural physiological regulation of the head position to improve breathing.

Significant mandibular setback was observed even 6 weeks after surgery (T3), when the hyoid bone showed an upward movement that was 82% of the immediate postoperative downward movement and TH showed movement that reversed 85% of the immediate setback. At T4, the hyoid bone and TH accounted for approximately 38% and 21% of the mandible setback, respectively. In response to the inadequate setback of the hyoid bone and tongue, the volume of the tongue caused its elevation to compensate for the constriction of the pharyngeal airway space. This phenomenon was reflected by the significant increase in the tongue depth at T2 and T4. With regard to changes in the position of the fourth vertebra, no significant changes were observed from T3 to T4, although it remained in a backward position.

Researchers [8, 9] have pointed out that the hyoid bone and its attached muscles play an important role in maintaining a normal pharyngeal airway size, because the location of the hyoid bone can change on the basis of different mandibular positions. They showed that mandibular advancement surgery can move the hyoid bone forward and enlarge the pharyngeal airway space, while mandibular setback can decrease the pharyngeal airway space. Surgery for mandibular deformity can change not only the patterns of bone and facial soft tissue but also the size of the pharyngeal airway space. Furthermore, mandibular setback surgery may cause OSA, which has already been reported by Riley et al. [10]. OSA syndrome (OSAS) refers to airway narrowing or collapse while sleeping, resulting in recurrent apnea that lasts for >10 s each time. According to the study, the oropharyngeal and hypopharyngeal spaces are closely related to the anatomical position of the mandibular bone, tongue, and hyoid bone. During backward movement of the mandibular bone, surgeons must clearly understand whether this structural change and hypopharyngeal space decrease can cause patient dyspnea or apnea. In particular, surgeons scheduled to operate on patients with OSA or facial deformity should assess the pharyngeal airway spaces of these patients before surgery.

According to a study by Muto et al. [11], mandibular setback using bilateral sagittal split osteotomy (BSSO) resulted in mean decreases of 2.6 and 4.0 mm in the pharyngeal airway space in the retropalatal and retroglossal regions, respectively. Furthermore, Tselnik and Pogrel [12] demonstrated that a mean mandibular setback of approximately 9.7 mm caused an approximate 12.8% decrease in the pharyngeal airway space. A study by Wenzel et al. [13] indicated that mandibular setback surgery resulted in a mean length decrease of 2 mm in the pharyngeal airway; however, its patency could still be maintained by adaptation to the situation. In our study, there was an immediate postoperative decrease of 0.9 mm (−0.9/23.2, −4%) in the NSP length, 4.1 mm (−4.1/21.5, −19.1%) in the UOP length, 1.7 mm (−1.7/15.9, −10.7%) in the LOP length, and 2 mm (−2/18, −11.1%) in the LGP length. Therefore, our patients showed a greater decrease in the retropalatal pharyngeal airway space compared with those in the abovementioned studies. Even at T4, a significant decrease of 4.7 mm (−4.7/21.5, −21.9%) and 2.4 mm (−2.4/15.9 = −15.1%) was observed in the UOP and LOP lengths, respectively. Because of postoperative airway narrowing, Marşan et al. [14] and Gu et al. [15] reported significant increases of 3.7° and 5.9° in the craniovertebral angle. Muto et al. [16] investigated the relationship between the craniocervical inclination and pharyngeal airway space and found that changes in the craniocervical inclination caused by head extension were correlated with an increase in the pharyngeal airway space. Our findings showed similar results; the craniocervical angle was significantly increased by 2.8° immediately after surgery. A raised head posture was observed in response to relevant structural and physiological changes after surgery.

Pearson's correlation analyses were performed to determine the correlation of immediate postoperative length decreases in NSP, UOP, LOP, and LGP with other immediate postoperative changes. No significant correlations were found between the NSP length and related factors. The amount of mandibular setback revealed no significant correlations with the length decrease in any segment of the pharyngeal airway space. The amount of hyoid bone setback influenced the decrease in the UOP and LOP lengths, and the amount of fourth cervical vertebral setback and an increase in the craniovertebral angle were significantly correlated with the decrease in the UOP, LOP, and LGP lengths. Even the tongue lengths and depths had changed, although there was no significant effect on the pharyngeal airway space. Further correlation analyses were conducted for the length decreases in NSP, UOP, LOP, and LGP at T4. No significant correlations were found between the NSP length and related factors, except UOP. The final mandibular position significantly influenced only the LGP length. The fourth cervical vertebra remained in a backward position, and an increase in the craniocervical angle still showed a significant correlation with decreased UOP and LOP lengths. These results further indicated that the pharyngeal airway space decreased after surgery, although the pharyngeal airway patency was adequately maintained through natural physiological adaptation.

Backward movement of the mandible will inevitably cause airway narrowing, which depends on not only the amount of setback but also the extent of soft tissue edema after surgery. Because of gravity, the throat and retrolingual space are narrower in the supine position than in the sitting or standing position. However, during sleep, the muscles of the nasal cavity, throat, and laryngeal pharynx may relax and the muscular tension may decrease. Consequently, the airway space will be much narrower during this time than during the awake state, causing partial airway obstruction, and can easily cause breathing problems. In particular, during the first night after surgery, the muscle relaxing effects of general anesthesia may persist, facilitating airway blockage. In IVRO, because the maxilla and mandible require fixation, patients cannot open their mouth; in addition, transnasal intubation will cause postoperative bleeding and edema in the nasal mucosa. For these reasons, particular attention should be given to patient safety.

In conclusion, our findings suggest that the oropharyngeal airway space is significantly decreased and correlated with a change in the head posture after mandibular setback surgery. The upper and lower segments of the oropharyngeal airway space were significantly shortened by mandibular setback surgery, except the nasopharyngeal and laryngeal airway spaces. An increase in the craniocervical angle was significantly correlated with length decreases in the upper and lower segments of the oropharyngeal airway space and the laryngeal airway space. This phenomenon reflects a natural physiologic response to a narrowed pharyngeal airway space.

## Figures and Tables

**Table 1:** Values for the various cephalometric parameters of the surgical changes in the horizontal and vertical direction.

Variable	T12			T23			T34			T14			T24		
	Mean	SD		Mean	SD		Mean	SD		Mean	SD		Mean	SD	
Horizontal (mm)															
Me	−12.8	4.60	∗	−3.3	5.29	∗	3.1	5.44	∗	−12.9	4.36	∗	−0.1	4.20	—
H	−4.9	7.30	∗	−1.5	7.61	—	1.7	7.23	—	−4.8	5.32	∗	0.2	6.27	—
TH	−7.1	6.87	∗	6.0	7.60	∗	−1.6	9.03	—	−2.7	8.04	∗	4.4	6.46	∗
C4	−5.6	8.67	∗	2.9	11.99	—	−0.3	7.43	—	−3.1	12.47	—	2.6	10.74	—
Vertical (mm)															
Me	1.6	5.24	—	−0.4	3.82	—	−1.5	7.33	—	−0.3	5.62	—	−1.9	6.75	—
H	10.7	5.79	∗	−8.8	4.48	∗	−0.2	8.84	—	1.7	7.97	—	−9.0	8.52	∗
TH	−2.7	7.53	∗	0.7	7.587	—	−2.6	7.10	∗	−4.6	6.41	∗	−1.9	8.40	—
C4	0.4	8.75	—	0.4	4.50	—	−1.2	9.27	—	−0.4	8.84	—	−0.8	8.95	—

^∗^Significant *p* < 0.05; —: not significant.

T12: postsurgical immediate change; T23: change between immediately and 6 weeks to 3 months after surgery; T34: change between 6 weeks to 3 months and over 1 year after surgery; T14: over 1-year final surgical change; T24: over 1-year surgical stability.

Me: menton; H: hyoid bone; TH: highest point on the tongue.

C4: fourth cervical vertebra.

**Table 2:** Linear distances and degree for the serial cephalometric parameters.

Variable	T12			T23			T34			T14			T24		
	Mean	SD		Mean	SD		Mean	SD		Mean	SD		Mean	SD	
Linear distance															
NSP	−0.9	2.71	—	1.6	3.27	∗	−0.3	2.73	—	0.4	2.53	—	1.2	3.07	∗
UOP	−4.1	4.42	∗	0.5	4.34	—	−1.1	4.56	—	−4.7	4.19	∗	−0.6	4.11	—
LOP	−1.7	4.59	∗	−1.7	3.61	∗	1.0	3.62	—	−2.4	3.22	∗	−0.7	4.55	—
LGP	−2.0	3.77	∗	1.5	3.80	∗	−0.2	3.63	—	−0.6	3.38	—	1.3	4.07	—
Tongue depth	8.0	5.14	∗	−4.6	6.04	∗	1.6	4.80	—	5.0	5.53	∗	−3.0	5.20	∗
Tongue length	0.7	5.43	—	−3.2	5.38	∗	0.0	5.63	—	−2.5	6.14	∗	−3.2	5.34	∗
H-E	1.3	5.09	—	−3.1	4.05	∗	1.2	4.37	∗	−0.5	5.16	—	−1.9	3.15	∗
Degrees															
C4-S-N	2.8	4.01	∗	−0.4	3.66	—	−0.6	4.44	—	1.7	5.16	—	−1.1	4.63	—

^∗^Significant *p* < 0.05; —: not significant.

T12: postsurgical immediate change; T23: change between immediately and 6 weeks to 3 months after surgery; T34: change between 6 weeks to 3 months and over 1 year after surgical; T14: over 1-year final surgical change; T24: over 1 year surgical stability.

NSP: nasopharyngeal airway; UOP: upper oropharyngeal airway.

LOP: lower oropharyngeal airway; LGP: laryngopharyngeal airway.

H-E: distance between hyoid bone and epiglottis.

C4-S-N: craniocervical angle.

**Table 3:** Relationship in the immediate postoperative changes (T12) between pharyngeal airway and various cephalometric parameters.

Variable	NSP	UOP	LOP	LGP
	CC	CC	CC	CC
Landmarks				
Horizontal				
Me	−0.168	−0.010	−0.091	−0.252
H	−0.205	−0.420^∗∗^	−0.270	−0.167
TH	−0.328^∗^	0.010	−0.053	−0.039
C4	−0.207	−0.577^∗∗^	−0.476^∗∗^	−0.286
Vertical				
Me	0.007	0.185	0.102	0.121
H	0.077	−0.018	0.081	−0.088
TH	0.138	0.225	−0.081	0.044
C4	0.005	−0.079	−0.168	−0.121
Linear (mm)				
NSP	1.000	0.179	0.021	0.032
UOP	0.179	1.000	0.610^∗∗^	0.360^∗^
LOP	0.021	0.610^∗∗^	1.000	0.445^∗∗^
LGP	0.032	0.360^∗^	0.445^∗∗^	1.000
H-E	−0.296	−0.076	−0.158	−0.054
Tongue depth	0.043	−0.275	0.106	0.102
Tongue length	0.307	0.305	−0.003	0.130
Degrees				
C4-S-N	0.193	0.647^∗∗^	0.504^∗∗^	0.265

CC: correlation coefficients.

^∗^Significant *p* < 0.05; ^∗∗^ *p* < 0.01.

NSP: nasopharyngeal airway.

UOP: upper oropharyngeal airway.

LOP: lower oropharyngeal airway.

LGP: laryngopharyngeal airway.

Me: menton; H: hyoid bone.

TH: highest point on the tongue.

C4: fourth cervical vertebra.

H-E: distance between hyoid bone and epiglottis.

C4-S-N: craniocervical angle.

**Table 4:** Relationship in the final postoperative changes (T14) between pharyngeal airway and various cephalometric parameters.

Variable	NSP	UOP	LOP	LGP
	CC	CC	CC	CC
Landmarks				
Horizontal				
Me	0.293	0.247	0.005	0.487^∗∗^
H	−0.035	−0.392^∗^	−0.279	−0.080
TH	0.002	−0.088	−0.044	0.142
C4	−0.129	−0.492^∗∗^	−0.378^∗^	−0.072
Vertical				
Me	0.011	0.005	−0.240	−0.225
H	−0.172	−0.122	−0.346^∗^	−0.319
TH	−0.065	0.174	−0.278	−0.017
C4	0.032	0.025	−0.051	−0.206
Linear (mm)				
NSP	1.000	0.332^∗^	0.200	0.243
UOP	0.332^∗^	1.000	0.484^∗∗^	0.405^∗^
LOP	0.200	0.484^∗∗^	1.000	0.313
LGP	0.243	0.405^∗^	0.313	1.000
H-E	0.043	−0.099	0.034	0.056
Tongue depth	−0.140	−0.223	0.000	−0.345^∗^
Tongue length	0.131	0.503^∗∗^	0.247	0.135
Degrees				
C4-S-N	−0.016	0.368^∗^	0.407^∗^	0.347^∗^

CC: correlation coefficients.

^∗^Significant *p* < 0.05; ^∗∗^ *p* < 0.01.

NSP: nasopharyngeal airway.

UOP: upper oropharyngeal airway.

LOP: lower oropharyngeal airway.

LGP: laryngopharyngeal airway.

Me: menton; H: hyoid bone.

TH: highest point on the tongue.

C4: fourth cervical vertebra.

H-E: distance between hyoid bone and epiglottis.

C4-S-N: craniocervical angle.
